# Comparative evaluation of antifungal susceptibility testing methods of invasive *Candida* species and detection of *FKS* genes mutations in caspofungin intermediate and resistant isolates

**DOI:** 10.1186/s12879-024-10435-8

**Published:** 2025-01-24

**Authors:** Dalia Saad ElFeky, Doaa Mahdy El-Wakil, Mai M. Mwafy, Mohamed M.A. Atia, Noha Mahmoud Gohar

**Affiliations:** 1 Department of Medical Microbiology and Immunology, Faculty of Medicine, Cairo University, Al-Saray Street, Al-Manial, Cairo, 11562 Egypt; 2 Department of Medical Microbiology and Immunology, Faculty of Medicine, Tanta University, Tanta, Egypt; 3 Genome Mapping Department, Agricultural Genetic Engineering Research Institute (AGERI), Agricultural Research Center (ARC), Giza, Egypt

**Keywords:** *Candida* species, ATB FUNGUS 3, Vitek-2 AST-YS08, Caspofungin, Acquired resistance, *FKS* mutations

## Abstract

**Background:**

Fungal invasive infections caused by *Candida* species pose a substantial public health risk with limited therapeutic options. Antifungal susceptibility testing (AFST) is necessary to optimize the therapy. The study aimed to compare different AFST methods of *Candida* spp. and detect *FKS* gene mutations among caspofungin-intermediate and resistant isolates.

**Methods:**

A total of 60 non-replicative invasive *Candida* isolates recovered from various clinical samples were included. In-vitro AFST was carried out using the ATB FUNGUS 3, Vitek-2 AST-YS08, and E-test. Hotspot (HS) regions of *FKS* genes were sequenced for caspofungin-intermediate and resistant isolates.

**Results:**

*Candida albicans* (58.3%) was the most predominant spp., followed by *C. glabrata* (28.3%). Based on the clinical breakpoints (CBPs), fluconazole resistance was found in *C. albicans* (45.7%), *C. tropicalis* (25%), and the *C. parapsilosis* isolate, while 35.3% of *C. glabrata* were susceptible dose-dependent (SDD). None of *C. albicans*, *C. tropicalis*, or *C. parapsilosis* isolates were resistant to voriconazole. Using the epidemiological cut-off values (ECVs) for amphotericin B, 6.7% of isolates were non-wild type (non-WT), including *C. guilliermondii* (50%), *C. tropicalis* (25%), and *C. glabrata* (11.8%), while all *C. albicans*, *C. parapsilosis*, and *C. kefyr* isolates were classified as wild-type (WT). ATB FUNGUS 3 and Vitek-2 had the highest categorical agreement (CA) (83.1%) for amphotericin B, while a lower concordance was detected with voriconazole (23.2%) and fluconazole (52.2%). For caspofungin, Vitek-2 and E-test had a CA of 89.8%. Eleven isolates (10 *C. glabrata* and one *C. parapsilosis*) exhibited resistance or intermediate susceptibility to caspofungin (MICs: 0.25‒>32 µg/ml). Molecular characterization of the *FKS* gene demonstrated that *FKS1* mutations V47I, V52K, V56T, D57S, L62F, I71Y, I71Q in the HS1 region, and G7S, P11H mutations in the HS2 region were associated with increased caspofungin MIC values (16 µg/ml). Mutations at the HS1 of the *FKS2* gene; K33V, W35K, and W35V; were associated with the highest caspofungin MICs of > 32 µg/ml.

**Conclusions:**

ATB FUNGUS 3 demonstrated acceptable performance for AFST, however, azole activity against *Candida* spp. should be interpreted carefully. Novel mutations within HS regions of *FKS* genes elucidated different levels of caspofungin resistance in *C. glabrata* and *C. parapsilosis* isolates.

**Supplementary Information:**

The online version contains supplementary material available at 10.1186/s12879-024-10435-8.

## Introduction

*Candida* species represent the primary cause of fungal infections, ranging from less severe forms of mucocutaneous candidiasis to fatal invasive illnesses [1]. *Candida albicans* typically causes the majority of *Candida* infections. However, the increasing usage of systemic antifungal medications globally for preventing or treating invasive fungal infections has resulted in increased infections attributed to non-albicans *Candida* (NAC) spp. [2].

Antifungal susceptibility testing (AFST) is necessary for managing cases with invasive fungal infections, those suspected of having acquired resistance, or those encountering refractory, relapsing, or breakthrough fungal infections [3]. Broth microdilution (BMD) is the preferred test for antifungal susceptibility in *Candida* spp. by determining the minimum inhibitory concentration (MIC) following the European Committee on Antimicrobial Susceptibility Testing (EUCAST) and the Clinical and Laboratory Standards Institute (CLSI) reference standards [4, 5]. However, BMD method is laborious, time-consuming, and challenging to apply in laboratories [6]. Several commercially available adaptations of the BMD method which facilitate the testing procedure and the interpretation of MIC results are currently available [7]. The Vitek-2 is a completely automated system that provides reproducible, rapid, and objective results for *Candida* spp. identification and MIC determination, showing excellent agreement with CLSI and EUCAST methods for flucytosine, amphotericin B, fluconazole, voriconazole [6, 8], and echinocandins [8]. The updated Vitek-2 AST-YS08 system (bioMérieux, France) was modified to align with the currently revised Clinical Breakpoints (CBPs) provided by CLSI for the prevalent spp. of *Candida* [9]. The ATB FUNGUS 3 (bioMérieux, France) is a simple, commercially available panel for the determination of MIC for *Candida* spp. with a reported high level of agreement compared to the BMD method [10], however, the absence of echinocandins in this panel restricts its practical utility in routine testing [3]. Another affordable alternative to the BMD is the use of gradient diffusion strips. The E-test is particularly suitable for resource-limited settings and offers the advantage of providing an MIC value that enhances the differentiation between true resistance and the trailing growth phenomenon [11].

Echinocandins including caspofungin, anidulafungin, and micafungin, are advised as the primary therapy for invasive candidiasis, targeting β-1,3-D-glucan synthase that is vital for synthesizing a critical cell wall component of fungi [12, 13]. Nevertheless, acquired echinocandin resistance among *Candida* spp. represent a significant factor contributing to therapeutic failure, often associated with point mutations occurring in specific regions known as hot spots (HS) of *FKS1* and *FKS2* genes encoding the subunits that catalyze the previous enzyme, leading to reduced drug sensitivity and higher MIC values [14, 15].

The EUCAST and CLSI established standardized AFST methods and CBPs to assess the MICs to echinocandins [3]. As per CLSI guidelines, isolates that exhibited caspofungin susceptibility could be reported as such, whereas confirmatory tests including anidulafungin, micafungin testing, or sequencing of *FKS* genes are recommended for caspofungin intermediate or resistance test results [16]. *FKS* mutations are a crucial clinical indicator of reduced response to therapy, therefore, molecular testing is the preferred method for detecting echinocandin resistance [17].

The study was conducted to evaluate the performance of various AFST methods in assessing the in-vitro activity of 5-flucytosine, amphotericin B, fluconazole, voriconazole, and echinocandins against different species of *Candida* and to investigate *FKS* gene mutations among caspofungin intermediate and resistant *Candida* isolates.

## Materials and methods

This study received approval from the Research Ethical Committee of the Institutional Review Board, Faculty of Medicine, Cairo University, Egypt [ID: N-166-2023] and was conducted per the guidelines outlined by the Declaration of Helsinki. The study included 60 non-replicate invasive *Candida* strains that had been previously recovered from different clinical samples (blood, endotracheal aspirates, pleural and peritoneal fluids) from patients admitted to a private hospital in Cairo, Egypt, during the period from January 2021 through December 2022. The isolates were preserved at ‒80 °C until further AFST at the Medical Microbiology and Immunology Department, Faculty of Medicine, Cairo University, Egypt.

### Subculture and identification of *Candida* isolates

All isolates were subcultured on Sabouraud dextrose agar (SDA) plates (Oxoid, UK), and incubated at 37 °C for 24 to 48 h aerobically. Conventional microbiological methods were used to identify *Candida* isolates, including colony morphology, Gram staining, and germ tube formation. Presumptive spp. identification was performed using the chromogenic agar medium (Himedia, India), then confirmed by the automated Vitek-2 system (YST ID cards, bioMérieux, France) following the instructions provided by the manufacturer.

### Antifungal susceptibility testing

Each *Candida* isolate was subjected to AFST to determine the MIC of different antifungal drugs using two commercial methods: ATB FUNGUS 3 strip (bioMerieux, France) and Vitek-2 AST-YS08 cards (bioMérieux, France). Susceptibility to echinocandins was assessed by the Vitek-2 AST-YS08 cards for caspofungin and micafungin, and E-test strips for caspofungin (Liofilchem, Italy).

### ATB FUNGUS 3 susceptibility method

The ATB FUNGUS 3 strip is composed of 16 pairs of cupules. The first pair served as a growth control and the subsequent 15 pairs contained 5 antifungal drugs with different concentrations, including 5-flucytosine, amphotericin B, fluconazole, itraconazole, and voriconazole. The test was carried out and interpreted following the manufacturer’s instructions.

### Vitek-2 fungal susceptibility testing using AST-YS08 cards

The Vitek-2 AST-YS08 card included serial dilutions of 6 antifungal drugs including amphotericin B, 5-flucytosine, fluconazole, voriconazole, caspofungin, and micafungin. The test was conducted according to the instructions provided by the manufacturer.

### E-test for caspofungin susceptibility testing

Caspofungin MICs (ranging from 0.002 to 32 µg/ml) were measured using E-test strips (Liofilchem, Italy) using ready-made RPMI agar (Liofilchem, Italy) “Ref. 11509”, following instructions provided by the manufacturers.

### Interpretation and analysis of AFST results

The interpretation of MIC readings was done according to CLSI by applying the species-specific CBPs for fluconazole, voriconazole, caspofungin, and micafungin [16], and the species-specific epidemiological cut-off values (ECVs) for itraconazole, amphotericin B and the following specie-drug combinations: *C. guilliermondii* and fluconazole, *C. kefyr* and fluconazole, *C. glabrata* and voriconazole, as well as *C. kefyr* and micafungin [18]. The CBPs were used to classify isolates into susceptible (S), intermediate (I), susceptible dose-dependent (SDD), or resistant (R) categories, while ECVs were applied to differentiate isolates into wild-type (WT) i.e., MIC ≤ ECV or non-wild-type (non-WT) i.e., MIC > ECV (Table S1). Due to the lack of CLSI CBPs or ECVs for 5-flucytosine on all spp., voriconazole with *C. guilliermondii* and *C. kefyr*, itraconazole with *C. albicans*, the MIC results of these agents were interpreted following the ATB FUNGUS 3 manufacturer’s instructions. Additionally, the obtained MIC value when testing caspofungin with *C. kefyr* was not interpreted, owing to the currently unavailable CBPs or ECVs.

Several published studies have demonstrated that the Vitek-2 method and the reference BMD described by the CLSI had excellent essential agreement (EA) (85‒100%) and categorical agreement (CA) (85.7‒99%) [6, 19, 20]. Thus, in the current work, the Vitek-2 was chosen as the gold standard test for the comparative analysis of results provided by the ATB FUNGUS 3 for 5-flucytosine, amphotericin B, fluconazole, and voriconazole. Calculation of EA, CA, very major error (VME), major error (ME), and minor error (MIE) was done as described previously [10]. Since no CLSI CBPs or ECVs were established for 5-flucytosine on all spp., voriconazole with *C. guilliermondii* and *C. kefyr*, CA was not calculated. The EA and CA of the Vitek-2 method and E-test were calculated regarding caspofungin. The reported limitation of the Vitek-2 method with caspofungin according to the manufacturer’s instructions, hinders its reliability as a gold standard. Accordingly, only EA and CA were used to describe the Vitek-2 and E-test agreement.

### Molecular analysis of the HS1 and HS2 of the *FKS* genes

#### DNA extraction, PCR amplification, and products analysis

Molecular testing of *Candida* isolates that displayed caspofungin resistance or intermediate results using the Vitek method and E-test was conducted at the Genome Mapping Department, Agricultural Research Center (ARC), Giza, Egypt. DNA extraction from fresh *Candida* isolates was carried out by QIAamp DNA Mini Kit (Qiagen, Germany) following the instructions provided by the manufacturer. Conventional PCR was done using oligonucleotide primers designed for the amplification of the HS regions of *FKS1* [21] for *C. glabrata* and *C. parapsilosis*. Additionally, home-designed primers were used to amplify the HS regions of *FKS2* for *C. glabrata* (GenBank accession no. NC_006034.2) (Table 1). The PCR mixture included 12.5 µl of EmeraldAmp Max PCR Master Mix (Takara, Japan), 5 µl of DNA template, 1 µl of each primer of 20 pmol concentration, and 5.5 µl of water, with a final reaction volume of 25 µl. The PCR cycling conditions involved an initial denaturation step at 94˚C for 5 min., followed by 35 cycles of denaturation at 94˚C for 30 s., annealing at 57.5˚C for 30 s., and extension at 72˚C for 45 s., with a final extension step at 72˚C for 10 min. The amplification was done in an Applied Biosystems 2720 thermal cycler (Applied Biosystems, California, USA). PCR product separation was performed by electrophoresis on 1.5% agarose gel (Applichem, Germany, GmbH) in 1x TBE buffer at room temperature with gradients of 5 V/cm. Gel analysis involved loading 15 µl of products into each slot and determining amplicon sizes using a 100-bp DNA ladder (Fermentas, thermofisher, Germany). Gel photographing was done by the Gel Doc XR + Gel Documentation System (Bio-Rad Laboratories, Inc., Hercules, California, USA).

**Table 1:** Primers sequences, target gene, and amplicon size of *FKS1* and *FKS2* hot spot regions

Target gene	Candida Spp.	Primer	Primers sequences (5’‒3’)	Amplicon size (bp)	Reference
***FKS1*** **HS-1**	Universal primers	*FKS1*HS1F	AAT GGG CTG GTG CTC AAC AT	796	**(21)**
		*FKS1*HS1R	CCT TCA ATT TCA GAT GGA ACT TGA TG		
***FKS1*** **HS-2**	Universal primers	*FKS1*HS2F	AAG ATT GGT GCT GGT ATG GG	636	
		*FKS1*HS2R	TAA TGG TGC TTG CCA ATG AG		
***FKS2*** **HS-1**	*C. glabrata*	*FKS2*HS1F	GCTTCTCAGACTTTCACCG	748	GenBank accession no. NC_006034.2
		*FKS2*HS1R	CAGAATAGTGTGGAGTCAAGACG		
***FKS2*** **HS-2**	*C. glabrata*	*FKS2*HS2F	CGAATCCATTCTTTGTATTTACG	400	
		*FKS2*HS2R	AGATCTGGCCCCCATATAAA		

#### *FKS* genes sequence analysis

To characterize *FKS* gene mutations, purification of the amplified PCR products was carried out by QIAquick PCR product extraction kit (Qiagen, Valencia) following the instructions provided by the manufacturer. The Sanger method was employed for genomic DNA sequencing by the BigDye Terminator V3.1 cycle sequencing kit (Perkin-Elmer), then purification of the sequenced DNA was performed using a Centrisep spin column. The DNA sequences were provided by Applied Biosystems 3130 genetic analyzer (HITACHI, Japan), and an initial BLAST (Basic Local Alignment Search Tool) analysis [22] was conducted to determine the sequence identity to GenBank accessions. The obtained DNA sequences were compared to the HS regions of *FKS1* gene of *C. glabrata* ATCC 90030 (GenBank accession no. HM366442), the HS regions of *FKS2* gene of *C. glabrata* (GenBank accession no. NC_006034.2), the HS1 of *FKS1* gene of *C. parapsilosis* ATCC 22019 (GenBank accession no. FJ372629), and the HS2 of *FKS1* gene of *C. parapsilosis* (GenBank accession no. EU221325). For the sequence analysis, both nucleotides and their translation were generated using the Unipro UGENE software package (X1).

### Statistical methods

All statistical calculations were conducted by the Statistical Package for the Social Sciences (SPSS) version 26 for Microsoft Windows. The MIC values were statistically described in terms of range, median or 50th percentile (MIC_50_), and 90th percentile (MIC_90_). The frequency (count) and relative frequency (percentage) were applied for categorical data. Spearman’s correlation analysis was used to correlate the MIC values of antifungals obtained by the different AFST methods.

## Results

### Species distribution of the *Candida* isolates

This study included 60 non-replicate invasive *Candida* isolates obtained from different clinical samples: 30 isolates (50%) from blood, 21 (35%) from endotracheal aspirates, and 9 (15%) from sterile body sites, as peritoneal and pleural fluids. The isolates comprised 35 *C. albicans* (58.3%) and 25 NAC spp. (41.7%). *C. glabrata* was the prevalent NAC responsible for 28.3% (17/60) of the total isolates, then *C. tropicalis* (6.7%, 4/60), and *C. guilliermondii* (3.3%, 2/60). Only one isolate of *C. kefyr* and *C. parapsilosis* (1.7% each) was detected. Additionally, *C. albicans*, *C. glabrata*, and *C. tropicalis* were obtained from all clinical specimens, whereas *C. guillermondii*, *C. kefyr*, and *C. parapsilosis* were exclusively isolated from blood.

### Results of antifungal susceptibility testing methods

The CBPs categorized *Candida* isolates as S, I, SDD, or R, while ECVs distinguished isolates as WT (MIC ≤ ECV) or non-WT (MIC > ECV) (Table S1). When CBPs were used for interpreting azole resistance rates by the Vitek-2 method, 15 (45.7%) *C. albicans*, one (25%) *C. tropicalis*, and a single *C. parapsilosis* isolate showed resistance to fluconazole. Meanwhile, none of the *C. glabrata* isolates were susceptible, however, six (35.3%) exhibited SDD to fluconazole. In contrast, none of *C. albicans*, *C. tropicalis*, or *C. parapsilosis* exhibited resistance to voriconazole. The ECVs were applied to interpret the results of amphotericin B and itraconazole among different *Candida* spp. Regarding amphotericin B as tested by the Vitek-2 method, 4 (6.7%) isolates were categorized as non-WT as follows: one (50%) *C. guilliermondii*, one (25%) *C. tropicalis*, and two (11.8%) *C. glabrata*, whereas 35 (100%) *C. albicans* isolates, along with the single *C. parapsilosis* and *C. kefyr* isolates were classified as WT. Moreover, itraconazole testing by ATB FUNGUS 3 revealed that two *C. tropicalis* and one *C. guillermondii* (50%, each), six (35.3%) *C. glabrata*, and both *C. kefyr* and *C. parapsilosis* isolates exhibited the non-WT category. In addition, the ECVs were used to interpret fluconazole with *C. guilliermondii* and *C. kefyr* using ATB FUNGUS 3 and were classified as non-WT. In contrast, 16 (94.1%) *C. glabrat*a were detected as WT when tested for voriconazole by the Vitek-2 method (Table S1).

Owing to the absence of CLSI CBPs or ECVs for 5-flucytosine with all *Candida* spp. and certain species-drug combinations, MIC results were interpreted using ATB FUNGUS 3 manufacturer guidelines. The overall resistance rate of 5-flucytosine was 1.7%, observed in a single *C. glabrata* isolate. A single *C. guilliermondii* and *C. kefyr* displayed resistance to voriconazole, while 30 (85.7%) *C. albicans* showed resistance to itraconazole.

Caspofungin resistance rates among all *Candida* spp. were interpreted using the spiece-specific CBPs. The overall resistance rate to caspofungin as measured by the E-test was 16.7%, with 6.6% showing intermediate susceptibility. Resistance to caspofungin was reported in *C. glabrata* (52.9%) and the single *C. parapsilosis* isolate. The two *C. guilliermondii* isolates exhibited intermediate susceptibility to caspofungin, whereas *C. albicans* and *C. tropicalis* displayed no resistance. Currently, no CBP or ECV has been established for caspofungin with *C. kefyr*. The E-test MIC result of this isolate was 0.25 µg/ml (Table S1).

Regarding echinocandins susceptibility testing by the Vitek-2 method, the overall resistance to caspofungin was 15%. Notably, there was one *C. kefyr* isolate for which the sensitivity to caspofungin remained unknown. For micafungin, the AST-YS08 was unable to provide susceptibility results for 13 isolates (10 *C. glabrata*, one *C. albicans*, one *C. parapsilosis*, and one *C. kefyr*). The remaining 47 isolates were determined to be sensitive to micafungin, as interpreted using CBPs, exhibiting MIC readings from ≤ 0.06–0.25 µg/ml. It was observed that 10 isolates with missed micafungin susceptibility results displayed resistance (9 *C. glabrata*) or intermediate (one *C. parapsilosis*) susceptibility to caspofungin. On the other hand, two isolates (one *C. glabrata* and one *C. albicans*) with missed micafungin susceptibility results were found to be susceptible to caspofungin. Therefore, for all caspofungin-resistant or intermediate susceptible isolates by the Vitek, no susceptibility data for micafungin are available except for a single isolate of *C. glabrata* that was sensitive to micafungin (MIC ≤ 0.06 µg/ml), but showed intermediate sensitivity to caspofungin.

Of note, the Vitek machine did not provide susceptibility results for the following species-drug combinations: *C. albicans* with micafungin (2.9%, 1/35), *C. glabrata* with amphotericin B (5.9%, 1/17), fluconazole (64.7%, 11/17), voriconazole (5.9%, 1/17), and micafungin (58.8%, 10/17), the two *C. guilliermondii* isolates with fluconazole (100%), and the single *C. kefyr* isolate with fluconazole, micafungin, and caspofungin, as well as the single *C. parapsilosis* isolate and micafungin.

### Comparative evaluation of antifungal susceptibility testing methods

#### Essential agreement

Table S1 describes the MIC ranges, MIC_50_, and MIC_90_ results of *Candida* spp., along with the EA and CA rates between ATB FUNGUS 3 and Vitek-2 systems for amphotericin B, 5-flucytosine, fluconazole, and voriconazole, as well as between Vitek-2 and E-test for caspofungin. Noteworthy, EA and CA were calculated based on the available results provided by the Vitek-2 method, and all missed data were excluded from the comparative analysis. Regarding the overall EA, the ATB FUNGUS 3 and Vitek-2 methods showed the highest concordance for 5-flucytosine (96.7%), followed by amphotericin B (86.4%), while a lower concordance was reported with voriconazole (23.7%) and fluconazole (19.6%). The ATB FUNGUS 3 method yielded a two-fold higher MIC_90_ values for amphotericin B (4 µg/ml) compared to the Vitek-2 method (1 µg/ml), while the 5-flucytosine MIC_90_ values were found to be ≤ 4 µg/ml by ATB FUNGUS 3 and ≤ 1 µg/ml by Vitek-2 method. We observed that the ATB FUNGUS 3 method showed higher MIC_90_ values for fluconazole (> 128 µg/ml) and voriconazole (> 8 µg/ml) in all *Candida* isolates compared to the Vitek-2 method (8 µg/ml and ≤ 0.12 µg/ml, respectively). Concerning caspofungin, the Vitek-2 method showed lower MIC_90_ values (0.5 µg/ml) when compared to the E-test (4 µg/ml).

All *Candida* spp. showed excellent EA with 5-flucytosine (94.1‒100%) and amphotericin B (91.4‒100%), while a lower rate was observed with amphotericin B and *C. glabrata* (76.5%), and no EA was reported with *C. guilliermondii* isolates. Additionally, a lower EA rate was reported with fluconazole and voriconazole, especially with *C. albicans* (20% and 14.3%, respectively) and *C. glabrata* (0% and 43.8%, respectively). For caspofungin, the Vitek-2 method and E-test showed an overall EA of 86.4% and the highest rate found with *C. albicans* (97.1%), *C. glabrata* (82.4%), and *C. tropicalis* (75%) (Table S1).

#### Categorical agreement

**Fig. 1:**
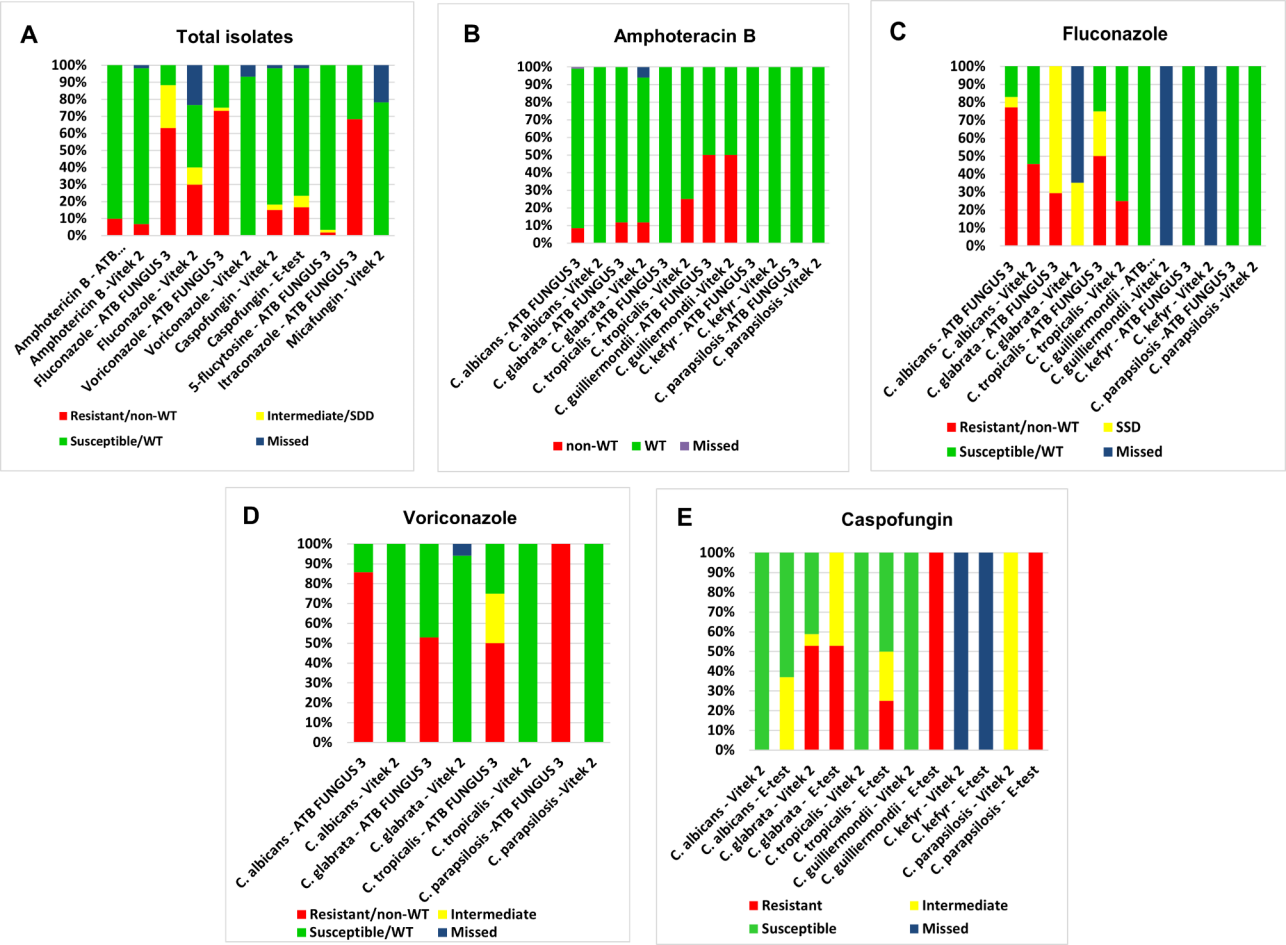
Comparative analysis between different antifungal susceptibility testing methods (AFST). **(A)** The ATB FUNGUS 3 and Vitek-2 results of amphotericin B, fluconazole, and voriconazole as well as the Vitek 2 system and E-test for caspofungin, the ATB FUNGUS 3 for 5-flucytosine and itraconazole and the Vitek-2 results of micafungin among the total *Candida* isolates. **(B)** Amphotericin B, **(C)** Fluconazole, **(D)** Voriconazole, and **(E)** Caspofungin results among different *Candida* spp

Regarding the overall CA, the ATB FUNGUS 3 and Vitek-2 methods showed the highest concordance for amphotericin B (83.1%), while a lower concordance was reported with voriconazole (23.2%) and fluconazole (52.2%) (Fig. 1A). The comparative analysis between ATB FUNGUS 3 and Vitek-2 systems for amphotericin B, fluconazole, and voriconazole, as well as the Vitek-2 and E-test for caspofungin among the different *Candida* spp. were shown in Fig. 1B‒1E. All *Candida* spp. showed good agreement with amphotericin B (75‒100%), while no CA was reported with *C. guilliermondii* isolates.

Analysis of VME of ATB FUNGUS 3 revealed 4 isolates (6.7%) with amphotericin B (two *C. glabrata*, one *C. tropicalis*, and one *C. guilliermondii*) and one isolate (2.2%) with fluconazole (*C. albicans*), with no VME was detected with voriconazole. In comparison, a high ME rate was detected in 42 (75%) isolates with voriconazole (30 *C. albicans*, 9 *C. glabrata*, two *C. tropicalis* and one *C. parapsilosis*) and 14 (30.4%) isolates with fluconazole (12 *C. albicans*, two *C. tropicalis*) (Table S1).

The Vitek-2 method and the E-test showed an overall CA rate of 89.8% for caspofungin, with *C. tropicalis* having the highest rate (100%), then *C. albicans* (97.1%) and *C. glabrata* (88.2%). It is worth mentioning that 6 (10.2%) isolates exhibited discrepancies between the two methods. The Vitek-2 method revealed intermediate susceptibility for two isolates (one *C. glabrata* and one *C. parapsilosis*), whereas the E-test identified them as resistant. On the other hand, among the four isolates that displayed intermediate susceptibility by the E-test, three were categorized as susceptible (one *C. albicans* and two *C. guillermondii*), whereas one isolate of *C. glabrata* was resistant by using the Vitek-2 method.

### Correlation analysis of antifungal susceptibility testing methods

**Fig. 2:**
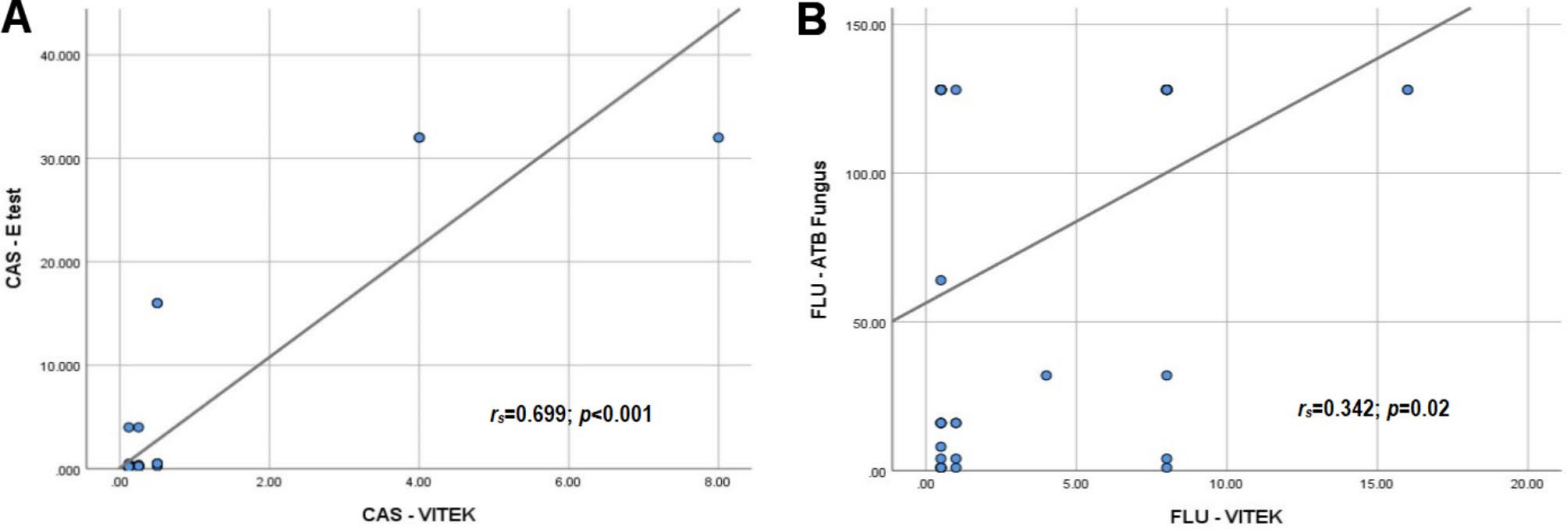
Scatter plot graphs showing the MIC values with **(A)** a strong correlation between the caspofungin-Vitek and caspofungin-E-test and **(B)** a weak correlation between fluconazole-Vitek and fluconazole-ATB FUNGUS 3 against *Candida* spp

Spearman’s correlation was applied to investigate the relationship between the different AFST techniques in measuring the MIC values of 5-flucytosine, amphotericin B, fluconazole, voriconazole, and caspofungin against various *Candida* spp. A strong correlation was observed between caspofungin-Vitek and caspofungin-E-test (*r*_*s*_=0.699; *p* < 0.001) (Fig. 2A), while a weak correlation was detected between fluconazole-Vitek and fluconazole-ATB FUNGUS 3 (*r*_*s*_=0.342; *p* = 0.02) (Fig. 2B).

### *FKS* hot spot mutations of *C. Glabrata* and *C. parapsilosis* isolates

The Vitek-2 and E-test methods revealed that 11 of the investigated *Candida* isolates (10 *C. glabrata* and one *C. parapsilosis*) were resistant or showed intermediate susceptibility to caspofungin. The susceptibility to micafungin for these isolates was undetermined, except one *C. glabrata* reported as susceptible. Regarding *C. glabrata*, the Vitek-2 method yielded caspofungin MIC values between 0.25 to ≥ 8 µg/ml, while the E-test method yielded caspofungin MIC values from 0.25 to > 32 µg/ml. Interestingly, isolates no. D83 and D225 showed the highest MIC readings (> 32 µg/ml) according to the E-test results. The MIC reading of *C. parapsilosis* was 4 µg/ml using the Vitek-2 system and > 32 µg/ml using the E-test (Table 2). These isolates were subjected to conventional PCR and sequencing of HS regions of *FKS1* (for *C. glabrata* and *C. parapsilosis*) and *FKS2* genes (for *C. glabrata*) to detect *FKS* mutations.

**Table 2:** *Candida* isolates harboring mutations within the *FKS* genes with MIC values of caspofungin

*Candida* spp.	Sample No.	Isolate	Caspofungin				Micafungin*	*FKS1* gene		*FKS2* gene	
			E-test	Category	Vitek-2	Category					
								HS1	HS2	HS1	HS2
***C. glabrata***	1	D24	0.25	I	0.5	R	ND	V47X, V50H, V52F, V56Q, D57Y	-	K33L, W35F	A90I
	2	D419	16	R	0.5	R	ND	I71Q	G7S, P11H	K33T, W35F	A90I
	3	D44	16	R	0.5	R	ND	V47I, V52K, V56T, D57S. L62F, I71Y	G7V	W44S	A90V
	4	D525	0.38	R	0.25	I	S	-	-	K33S, W35S	A90I
	5	D83	> 32	R	4	R	ND	-	-	K33V, W35K	A90I
	7	D225	> 32	R	≥ 8	R	ND	-	-	W35V	A90V
	8	D303	0.5	R	0.5	R	ND	-	G7V	W35S	A90V
	9	D305	0.5	R	0.5	R	ND	L62S	G7V	-	A90I
	10	D310	0.5	R	0.5	R	ND	-	-	-	A90I
	11	D417	0.5	R	0.5	R	ND	-	G7V	W35S	-
***C. parapsilosis***	6	D131	> 32	R	4	I	ND	-	S11P	-	-

Abbreviations: ND, Not determined; HS, Hot Spot; S, susceptible; I, intermediate; R, resistant

*Micafungin susceptibility was examined by the Vitek-2 system. Data was not given by the Vitek machine, except isolate D525 reported as susceptible

### Molecular characterization of the amino acid substitutions within *FKS* genes

**Fig. 3:**
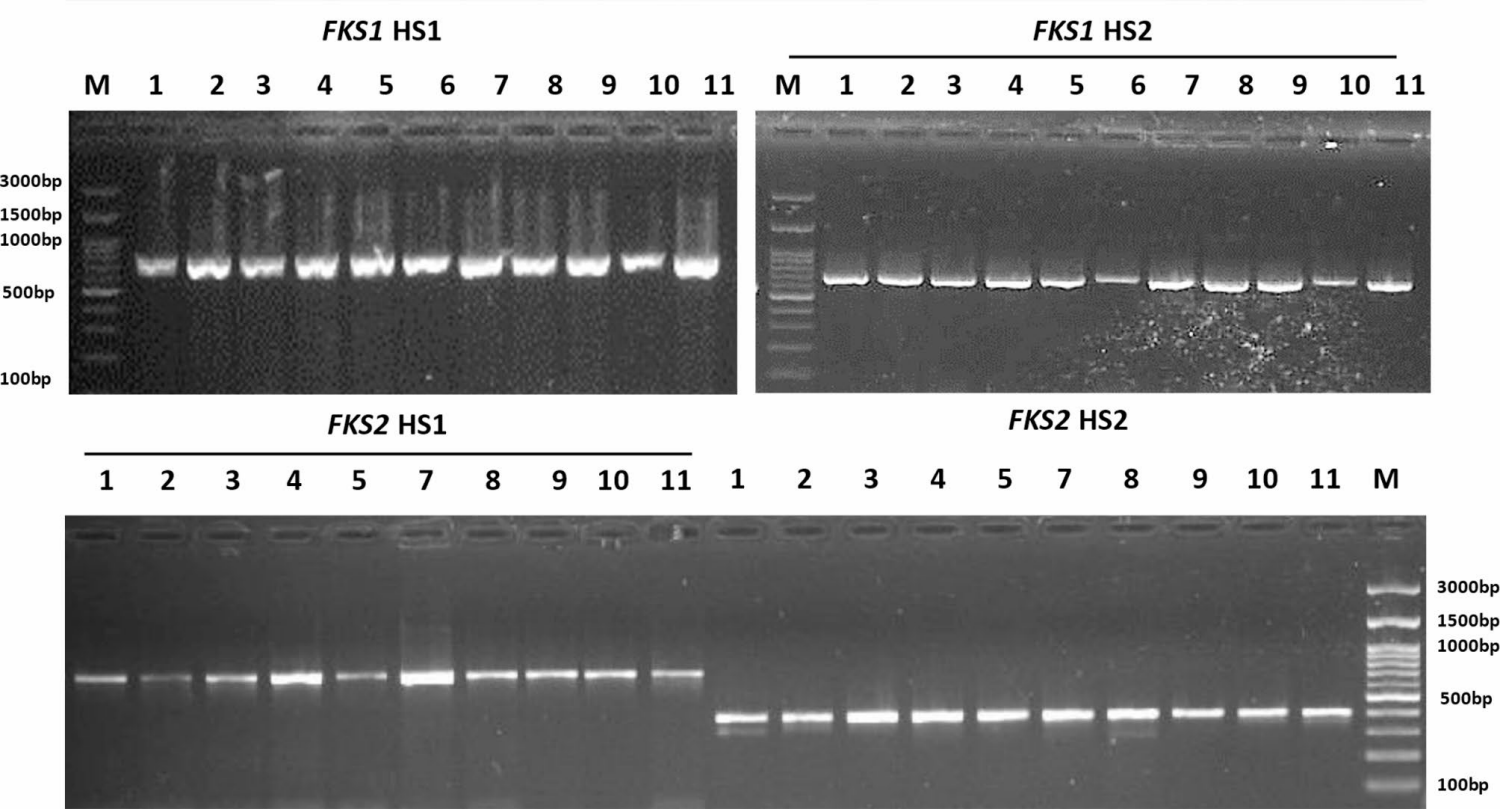
Gel electrophoresis showing the amplified products of *FKS1*-HS1, *FKS1*-HS2, *FKS2*-HS1, and *FKS2*-HS2 regions of *Candida* isolates. Lane M: DNA ladder, Lanes 1,2,3,4,5,7‒11: the *FKS1* gene amplicon of *C. glabrata* isolates. Lan 6: the *FKS1* gene amplicon of *C. parapsilosis* isolate

**Fig. 4:**
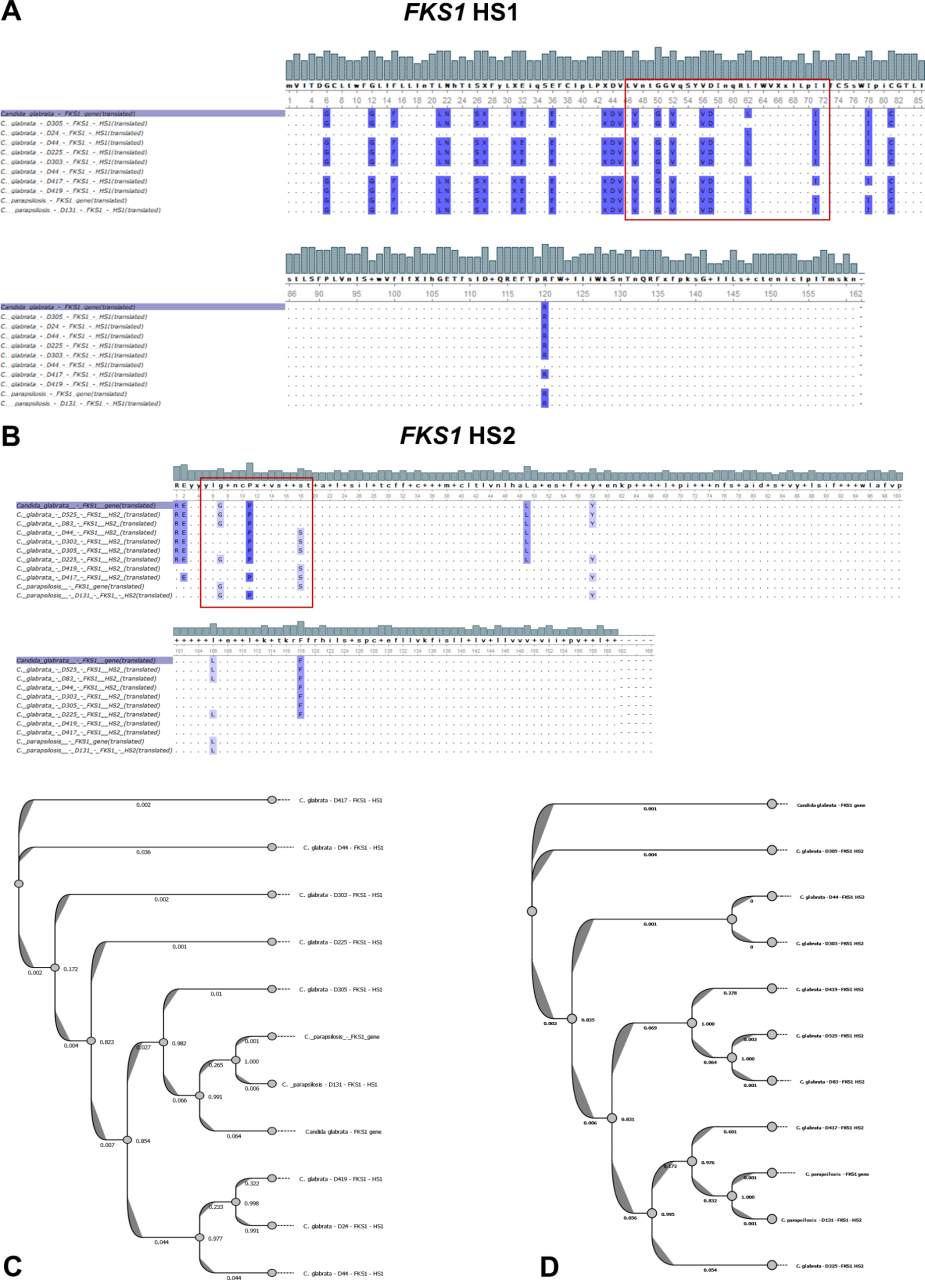
Multiple amino acid sequence alignments of **(A)*** FKS1*-HS1 and **(B)*** FKS1*-HS2 regions of *Candida* isolates that harbor mutations against the reference *FKS1* gene. Additionally, cluster analysis dendrograms showing the overall similarities at the nucleotide level of **(C) ***FKS1-*HS1 and **(D)*** FKS1*-HS2 of *Candida* isolates against the reference *FKS1* gene

**Fig. 5:**
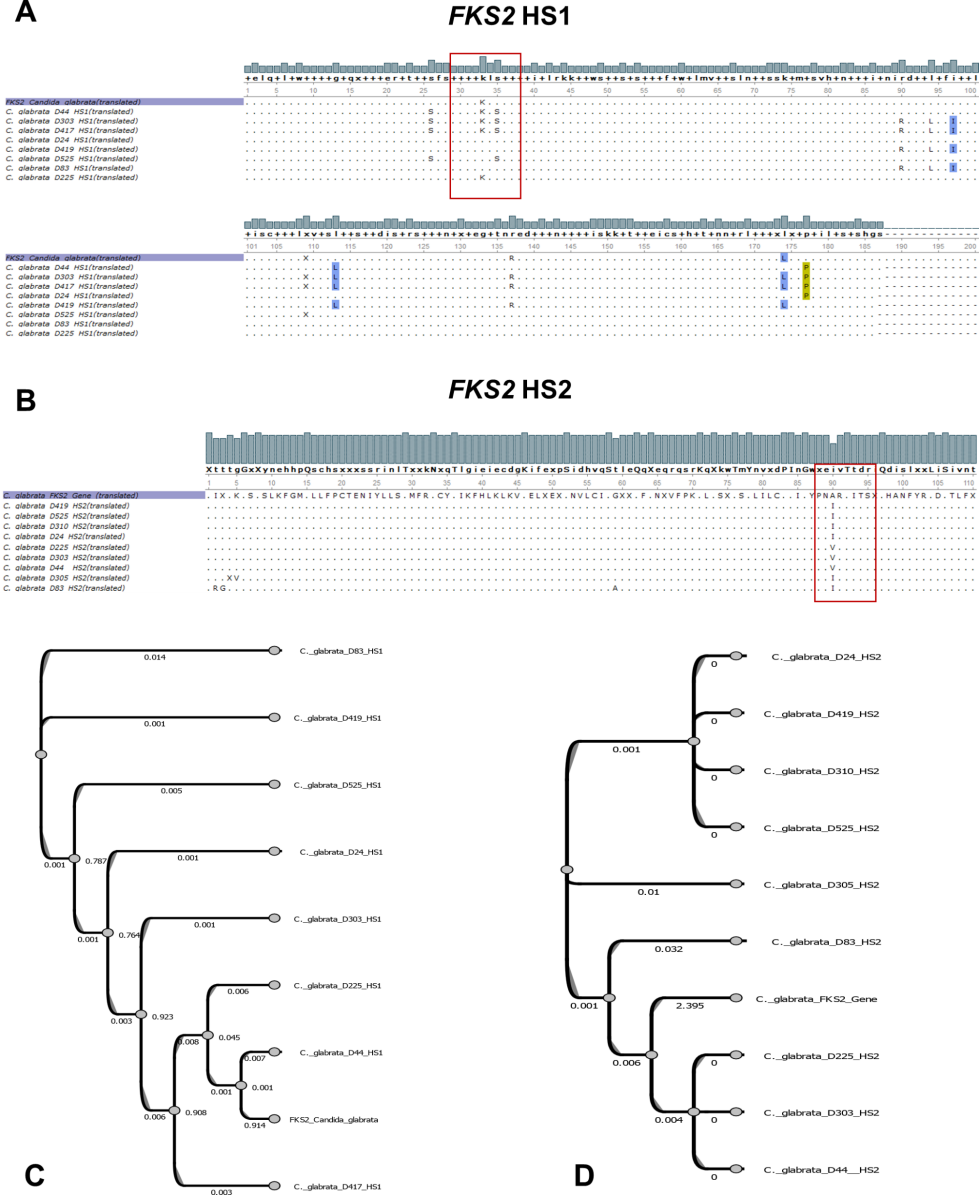
Multiple amino acid sequence alignments of **(A)*** FKS2*-HS1 and **(B)*** FKS2*-HS2 regions of *Candida* isolates that harbor mutations against the reference *FKS2* gene. Additionally, cluster analysis dendrograms showing the overall similarities at the nucleotide level of **(C)*** FKS2*-HS1 and **(D)*** FKS2*-HS2 regions of *Candida* isolates against the reference *FKS2* gene

The specific PCR results of the HS1 and HS2 for both *FKS1* and *FKS2* genes revealed a successful amplification of the target regions (Fig. 3). The Sanger sequencing results of the amplified products, *FKS1*-HS1, *FKS1*-HS2, *FKS2*-HS1, and *FKS2*-HS2, revealed many silent mutations at the nucleotide level within all sequenced isolates compared to the *C. glabrata* ATCC 90030 reference strain *FKS1* and *FKS2* genes, as well as the *FKS1* gene of *C. parapsilosis*. At the amino acid level, almost all the mutations generating amino acid substitutions in both *FKS* genes were found in both previously described mutational regions either for HS1 or HS2 or both (Figs. 4A and B, and Figs. 5A and B). Additionally, sequence alignment results revealed that almost all the 11 tested isolates harbor at least one or more mutations not previously implicated in the reference *C. glabrata* or *C. parapsilosis* strains. The HS2 within the *FKS2* in the *C. glabrata* isolates presented the most consistent amino acid substitution A90I, followed by the G7V substitution within the HS2 of *FKS1* in the *C. glabrata* isolates (Table 2). For *C. glabrata*, the isolates D525, D83, D225, and D310 showed no amino acid substitution mutational events within the two hot spots of the *FKS1* gene. For *C. parapsilosis*, only the HS2 of the *FKS1* gene showed a single amino acid substitution mutational event (S11P). It is worth noting that isolates D24 and D44 exhibited the highest occurrence of amino acid substitution mutations within the HS1 of the *FKS1* gene, with 5 and 6 events, respectively (Table 2). Ultimately, the dendrogram topology of the *FKS1*-HS1, *FKS1*-HS2, *FKS2*-HS1, and *FKS2*-HS2 of the analyzed isolates revealed a consistent clustering with the sequence alignment results (Figs. 4C and D, and Figs. 5C and D).

## Discussion

The increasing occurrence of invasive *Candida* infections globally has significant implications for mortality rates, hospital stays, and healthcare costs [23]. Fungi contribute to about 15% of healthcare-associated infections, with 70–90% of invasive candidiasis cases caused by *Candida* spp. [24]. Among *Candida* spp., *C. albicans*, *C. tropicalis*, *C. glabrata*,* C. parapsilosis*, and *C. krusei* being responsible for more than 90% of candidemia cases [12]. In the current work, *C. albicans* was the most prevalent spp. (58.3%), while NAC spp. accounted for 41.7%. Studies conducted in Egypt, Italy, and India revealed similar fundings, where *C. albicans* was also the predominant spp. obtained from respiratory tract samples [25], blood [26], and various clinical samples, including oropharyngeal swabs, blood, pus, and wound swabs [27]. However, other studies from Egypt, Turkey, Saudi Arabia, and China have observed a transition from the dominance of *C. albicans* to NAC spp. that were exclusively isolated from blood [28–32].

As regards NAC, our study found that the predominant spp. was *C. glabrata* (28.3%), then *C. tropicalis* (6.7%), and *C. parapsilosis* (1.7%). This result was in agreement with studies conducted in Nordic countries, France, the United States, and the ARTEMIS global surveillance study, where *C. glabrata* was also identified as the prevailing NAC spp. [33–36]. However, studies in Egypt reported *C. tropicalis* and *C. krusei* as the most isolated NAC spp. [25, 28, 29], while studies in Turkey and Italy found *C. parapsilosis* to be the predominant NAC spp. [26, 30]. The discrepancy in results between the different studies could be linked to geographical variations, the different sources of clinical samples, and host-related factors, including age, co-existing medical conditions like malignancies, surgical interventions, and the use of central venous lines.

Currently, other uncommon *Candida* spp., such as *C. guillermondii*, *C. kefyr*, and *C. lusitaniae* pose a rising concern to candidemia patients [28]. Our study reported two (3.3*%) C. guillermondii* isolates, which was in line with Lin et al. (1.2%) [32]. On the other hand, we observed only one isolate of *C. kefyr* (1.7%) in our hospital, a finding similar to a previous study from Egypt [28]. *C. kefyr* has been identified as a notable fungal pathogen, responsible for bloodstream infections, particularly in immunocompromised individuals with high mortality rates [28].

Despite the availability of many antifungal medications for *Candida* infection treatment, antifungal resistance is a major contributor to unsuccessful therapy and increased mortality rates [29]. Our study found fluconazole resistance in 45.7% of *C. albicans*, 25% of *C. tropicalis*, and the single *C. parapsilosis* isolates. Variable resistance patterns were recorded in previous studies conducted in Egypt, where fluconazole resistance rates were 41.8%, 27.9%, and 13.1% with the predominance of *C. tropicalis* (45.5%), *C. krusei* (100%), and *C. glabrata* (66.7%), respectively [25, 29, 31]. Notably, none of the *C. glabrata* isolates were susceptible, however, 35.3% exhibited SDD to fluconazole. Similarly, an Indian study reported that none of *C. glabrata* isolates were susceptible to fluconazole, with 79% categorized as SDD [30]. The high rates of fluconazole resistance observed in our study may be attributed to their extensive usage and/or inappropriate treatment practices. Regarding voriconazole, none of *C. albicans*, *C. tropicalis*, or *C. parapsilosis* exhibited resistance, and most *C. glabrat*a isolates (94.1%) were detected as WT, making it a valuable treatment option in situations where resistance to fluconazole is a significant concern. Consistent with our results, a previous study observed the absence of voriconazole resistance among *C. albicans* and *C. tropicalis*, whereas, 5% of *C. parapsilosis* were resistant and 57% of *C. glabrata* were classified as non-WT [30]. By contrast, El-Ganiny et al. reported voriconazole resistance in 6 (8.6%) *C. albicans*, 3 (12%) *C. krusei*, and one (12.5%) *C. tropicalis* [25].

For amphotericin B, 6.7% of *Candida* spp. were categorized as non-WT, mainly in *C. guilliermondii* (50%), *C. tropicalis* (25%), and *C. glabrata* (11.8%), while all *C. albicans*, *C. parapsilosis*, and *C. kefyr* isolates were classified as WT. In a comparison, Yenişehirli et al. reported a higher prevalence of non-WT for amphotericin B (16%), with the highest rate in *C. glabrata* (29%), followed by *C. tropicalis* (15%), *C. parapsilosis* (14%), and *C. albicans* (12%) [30]. Our findings suggest that most *Candida* spp. remain susceptible to amphotericin B, making it an effective option for treating fungal infections, particularly against azoles and echinocandins-resistant *Candida* strains. However, reduced susceptibility to *C. guilliermondii*, *C. tropicalis*, and *C. glabrata* emphasizes the need for routine AFST to ensure effective treatment.

In our study, the Vitek-2 method was used as the gold standard in evaluating the performance of ATB FUNGUS 3 with 5-flucytosine, amphotericin B, fluconazole, and voriconazole. Our study showed the highest overall EA with 5-flucytosine (96.7%). A good EA and CA for amphotericin B (86.4% and 83.1%) was observed, while lower rates were reported with fluconazole (19.6% and 52.2%) and voriconazole (23.7% and 23.2%), particularly with *C. albicans* and *C. glabrata*. Additionally, a high ME rate was detected with voriconazole (75%) and fluconazole (30.4%). Limited research has been conducted to assess the efficacy of ATB FUNGUS 3 in comparison to the BMD method. In line with our findings, Zhang et al. assessed the performance of visual readings of ATB FUNGUS 3, identifying the highest levels of agreement with 5-flucytosine and amphotericin B. However, contrary to our results, they reported higher levels of agreement with fluconazole and voriconazole [10]. Likewise, additional research has assessed ATB FUNGUS 2, an earlier version of ATB FUNGUS, in comparison with the CLSI method and found a higher agreement rate for amphotericin B and 5-flucytosine by both visual and automated readings, but a lower rate with fluconazole and voriconazole using automated reading [37]. Despite the simplicity and precision of the ATB FUNGUS 3 visual reading technique for MIC determination against *Candida* spp. [10], the occurrence of MEs in azole drug susceptibility evaluated by the ATB FUNGUS 3 in the present work can lead to inappropriate selection of clinical antifungal drugs and falsely elevated rates of antifungal resistance.

During the period starting in 2000, when echinocandins were introduced for clinical use, it was widely believed that resistance to this family of antifungal drugs would be uncommon [38]. In our study, we have assessed caspofungin susceptibility by the Vitek-2 and E-test methods. Our results revealed that the overall resistance rate to caspofungin among *Candida* spp. was 16.7% by the E-test and 15% by the Vitek-2 method. Lower resistance rates ranging from 1 to 4.1% were observed in different studies [25, 29, 31, 39]. While echinocandin resistance remains rare among *Candida* spp., there have been reports of increased MIC values after exposure to echinocandins, especially *C. glabrata* [40]. In our study, caspofungin resistance was observed with 52.9% of *C. glabrata* isolates and the single *C. parapsilosis* isolate, whereas no resistance was reported in *C. albicans* and *C. tropicalis*. Likewise, prior investigations have demonstrated the absence of caspofungin resistance among *C. albicans* [25, 26]. Contrary to our results, Mencarini et al. [26] reported a lower incidence of caspofungin resistance (1.9%) among *C. glabrata*, while previous research indicated that resistance of *C. glabrata* to echinocandins ranged from 3 to 13% [41]. In our study, micafungin susceptibility was observed in 78.3% of isolates, while there were 13 isolates for which the sensitivity to micafungin remained unknown. Likewise, high overall micafungin susceptibility rates were reported in Turkey (92.4%) [20] and Ethiopia (96%) [39], while a study from South Korea reported a susceptibility rate of 85.9% with *C. glabrata* [9].

Irrespective of the *Candida* spp., the Vitek-2 method yielded lower MIC_90_ values with caspofungin (0.5 µg/ml) than the E-test (4 µg/ml). This discrepancy is due to differences in the accuracy and performance between the two methods. For caspofungin susceptibility, no studies have assessed the Vitek-2 and E-test agreement, however, a previous Indian study demonstrated that the CLSI BMD and E-test for caspofungin testing yielded comparable MIC results, while the Vitek-2 method exhibited minor variations that were not statistically significant [42]. Similarly, a study from Austria demonstrated that the E-test had higher MIC_90_ values for caspofungin (0.354 µg/ml) compared to the EUCAST (0.063) and Sensititre YeastOne (0.120 µg/ml) methods [43]. In contrast, a study conducted on 133 isolates of *Candida* spp. revealed that the E-test MICs for caspofungin were two-fold dilution less than those measured with the CLSI [44]. Regarding caspofungin in this work, we observed that the Vitek-2 and E-test showed overall EA and CA of 86.4% and 89.8% respectively. *C. albicans* showed 97.1% EA and 97.1% CA, *C. glabrata* had lower EA and CA rates of 82.4% and 88.2%, and *C. tropicalis* had EA and CA rates of 75% and 100%.

In our study, the Vitek machine did not show results with fluconazole for 14 isolates (11 *C. glabrata*, two *C. guilliermondii*, and *C. kefyr*). Consistent with our results, prior studies have noted that YS08 failed to yield MIC results with fluconazole for any of the *C. glabrata* isolates [45–47] As per the manufacturer’s instructions, the Vitek-2 AST-YS08 system (bioMérieux) advises employing an alternative method before reporting fluconazole with *C. glabrata* and *C. kefyr*. Additionally, the Vitek machine failed to give results for voriconazole and amphotericin B of one isolate of *C. glabrata*, meanwhile, the susceptibility of three isolates (two *C. guilliermondii* and one *C. kefyr*) to voriconazole was undetermined, owing to the absence of CBPs or ECVs defined by the CLSI.

Due to the significant interlaboratory variability in caspofungin MIC measurements using CLSI and EUCAST methods, it has been proposed that anidulafungin or micafungin may be better suited for testing resistance to echinocandin [48]. Alternatively, CLSI guidelines recommended confirmatory tests for caspofungin intermediate or resistance test results including micafungin or anidulafungin testing, or *FKS* genes sequencing [16]. Confirmation of echinocandin resistance occurs when an isolate demonstrates resistance to a minimum of two echinocandins or when it contains mutations in the HS region of the *FKS* gene [48]. However, one of the limitations mentioned by the manufacturer’s instructions for the Vitek AST-YS08 system is the inability to detect resistance with micafungin and *Candida* spp. In our study, there were 13 isolates for which the sensitivity to micafungin remained unknown including the 10 isolates (9 *C. glabrata* and one *C. parapsilosis*) that exhibit resistance or intermediate susceptibility to caspofungin. Consequently, micafungin susceptibility testing by the Vitek-2 method can not be utilized as a reliable confirmation in such cases. Hence, we relied on the detection of *FKS* mutations to confirm caspofungin resistance. In line with our finding, a prior study comparing micafungin susceptibility between the AST-YS08 and BMD methods revealed that the YS08 card failed to detect micafungin MICs of ≤ 0.03 µg/ml with *C. glabrata* [47].

Our findings demonstrated that the correlation between AFST methods varies significantly depending on the antifungal agent and the method used. The strong correlation between caspofungin-Vitek and caspofungin-E-test (*r*_*s*_=0.699) suggests that these methods provide consistent results for caspofungin, indicating their reliability and interchangeability in clinical practice. On the other hand, the weak correlation between fluconazole-Vitek and fluconazole-ATB FUNGUS 3 (*r*_*s*_=0.342) highlights potential discrepancies in fluconazole testing results, which may impact clinical decision-making.

In our study, molecular testing was conducted on isolates that exhibited either resistance or intermediate results to caspofungin according to both the E-test and Vitek 2 method. It has been reported that the E-test CBP value of 0.25 µg/ml demonstrated the highest sensitivity (100%) and specificity (94%) in identifying *C. glabrata* isolates with *FKS* mutations [40]. Six isolates showed discrepancies between the Vitek-2 and E-test methods, among them three isolates had *FKS* mutations. One *C. glabrata* (isolate no. D525) and one *C. parapsilosis* (isolate no. D131) demonstrated intermediate susceptibility by the Vitek-2 method, while classified as resistant by the E-test. Conversely, one isolate of *C. glabrata* (no. D24) was read as resistant by the Vitek-2 method but was read as intermediate by the E-test. Generally, we observed that in cases where the MIC value exceeded the susceptibility CBPs by at least two doubling dilutions, a related *FKS* mutation was found in either *FKS1* or *FKS2*. Exceptions were observed in isolates no. D24, D525, and D131 where the MICs of caspofungin were found to be within 2 doubling dilutions from the susceptibility CBPs. However, *FKS* mutations were detected in these isolates, a finding supported by our study and Fraser et al. [49].

In general, echinocandin resistance in clinical settings is commonly linked to amino acid substitutions occurring in the HS regions of *FKS1* in all species of *Candida*, and *FKS2* specifically for *C. glabrata* [15]. The majority of *Candida* spp. rarely exhibit echinocandin resistance, however, *C. glabrata* has demonstrated a higher propensity for such resistance [41]. In our study, the *FKS*-mutant *C. glabrata* isolates exhibited caspofungin MIC readings of 0.25‒≥8 µg/ml by Vitek-2 method and 0.25‒>32 µg/ml by the E-test. A study from Switzerland reported *FKS* mutations in 4 isolates of *C. albicans* and 5 *C. glabrata* making them non-susceptible to all echinocandins, with a reported higher caspofungin MIC of 0.25‒>16 µg/ml in comparison to anidulafungin or micafungin MICs [50]. Generally, the MIC data indicates that different *FKS* mutations do not result in equal levels of resistance. The *FKS1* mutations V47I, V52K, V56T, D57S, L62F, I71Y, and I71Q (at the HS1), G7S, and P11H (at the HS2) exhibit higher caspofungin MIC values (16 µg/ml) as detected by the E-test. Interestingly, the *FKS2* mutations K33T and W44S were associated with an MIC reading of 16 µg/ml to caspofungin, whereas K33V, W35K, and W35V mutations (at the HS1) resulted in increased caspofungin MICs of > 32 µg/ml. A previous Turkish study reported different mutations (D666V, S663P, and delF659) in *FKS2* HS1 of *C. glabrata* strains with high MICs for echinocandin [51]. In another surveillance study, elevated MIC values for one or more echinocandin drugs were detected in 16 *C. glabrata* isolates that exhibited a mutation in HS1 of either the *FKS1* (two isolates) or *FKS2* (14 isolates) gene, with S663P mutation in the *FKS2* gene being the most frequently detected [52]. Nonetheless, investigations have suggested that the detection of *FKS* mutation is a more reliable predictor of the echinocandin treatment outcome, rather than the MIC value [17].

Notably, one *C. glabrata* isolate (D525) harboring three mutational events in *FKS2* exhibited intermediate susceptibility to caspofungin by the Vitek-2 method, but showed a micafungin MIC reading of ≤ 0.06 µg/ml that was classified as micafungin-susceptible by the Vitek-2 system. It has been suggested that treatment failure can arise from *FKS* gene mutations in the absence of phenotypic resistance [17], with previous echinocandin treatment being the most significant risk factor for phenotypic resistance or gene mutations [53]. Thus, the *FKS* mutations are considered a more reliable indicator of the diminished sensitivity of *C. glabrata* to echinocandins and decreased treatment response [54].

Despite patients infected with *C. parapsilosis* responding well to echinocandin treatment, the risk of developing resistance increases with repeated exposure to echinocandins [55]. In this work, the MIC reading of *C. parapsilosis* was 4 µg/ml by the Vitek-2 system and > 32 µg/ml by the E-test. It was shown that *C. parapsilosis* exhibited inherently greater echinocandin MIC readings in comparison to other commonly found *Candida* spp., owing to the naturally occurring proline-to-alanine substitution (P660A) in the HS1 region of the *FKS1* gene [56]. We identified a novel strain of *C. parapsilosis* that exhibited resistance to caspofungin and harbored a single mutation (S11P) at the *FKS1* HS2 region, which could be linked to its acquired resistance to caspofungin. Previous studies have reported two mutations (F652S and S656P) in *FKS1* HS1 of pan-echinocandin-resistant *C. parapsilosis* isolates [57, 58]. Another recent study identified specific mutations in *FKS1*-HS1 (R658S) and *FKS1*-HS2 (L1376F) of *C. parapsilosis* that contribute to its echinocandin resistance phenotype [59]. To our knowledge, this is the first Egyptian study to discover a diverse range of distinct mutations within HS regions of *FKS* genes in *C. glabrata* and *C. parapsilosis*, elucidating different levels of caspofungin resistance, However, the functional role of these mutations remains to be verified.

## Conclusions

Our data suggest that 5-flucytosine, amphotericin B, and voriconazole have the highest efficacy among the tested antifungal agents. Conversely, fluconazole and itraconazole exhibited lower levels of activity. Despite its rapidity and automation, the Vitek AST-S08 system has some limitations, such as a restricted database, potential limitations in detecting resistance in certain species-drug combinations, and cost considerations not being accessible in limited resources laboratories. The ATB FUNGUS 3 was a promising method for susceptibility testing due to its simplicity, rapidity, and broad spectrum of antifungal coverage, with good overall agreement for 5-flucytosine and amphotericin B against *Candida* spp.; however, its reliability in detecting azole susceptibility, particularly with *C. albicans* and *C. glabrata*, is questionable, and additional studies using the gold standard CLSI BMD method are necessary to validate this issue. On the other hand, caspofungin resistance primarily driven by *FKS* mutations is emerging, particularly in *C. glabrata* and *C. parapsilosis*, due to the limited therapeutic options available. The newly identified mutations within HS regions of *FKS* genes in both *C. glabrata* and *C. parapsilosis* isolates might explain different caspofungin resistance levels. Further research is necessary to explore the role of these mutations in conferring resistance to various echinocandins and more focused species-specific analyses are recommended in future studies to further refine the understanding of drug resistance mechanisms in *Candida* spp.

## Electronic Supplementary Material

Below is the link to the electronic supplementary material.

Supplementary Material 1

## Data Availability

All datasets generated and/or analyzed during the current study were presented in the main manuscript and are available from the corresponding author upon reasonable request. Sequence data that support the findings of this study have been submitted to the GenBank with a BankIt ID of 2850287.

## Abbreviations

AFST: Antifungal susceptibility testing
BLAST: Basic local alignment search tool
BMD: Broth microdilution
CA: Categorical agreement
CBPs: Clinical Breakpoints
CLSI: Clinical and Laboratory Standards Institute
EA: Essential agreement
ECVs: Epidemiological cut-off values
EUCAST: European Committee on Antimicrobial Susceptibility Testing
HS: Hot spots
ME: Major error
MIC: Minimum inhibitory concentration
MIE: Minor error
NAC: Non-albicans Candida
VME: Very major error
