# Supplementary material for: Comparative evaluation of antifungal susceptibility testing methods of invasive Candida species and detection of FKS genes mutations in caspofungin intermediate and resistant isolates
Source: BMC Infect Dis. 2025 Jan 24;25:114. doi: 10.1186/s12879-024-10435-8 (PMC11760087; doi:10.1186/s12879-024-10435-8)
Supplement: Supplementary file 1 — Supplementary Material 1 [file 12879_2024_10435_MOESM1_ESM.docx]

**Table S1** *In-vitro* susceptibility testing of 60 isolates of *Candida* spp. as determined by ATB FUNGUS 3, Vitek 2 system, and E-test based on CBPs as per CLSI supplement M60 (16) or ECVs as per CLSI supplement M59 (18)

| ***Candida* species/ Antifungal agent** | **Method** | **CBPs/ECVs^a^**  **(μg/mL)** | **MIC**  **(μg/mL)^b^** | | | **EA^c^**  **No. (%)** | **No. (%) of isolates** | | | **CA^c^**  **No. (%)** | **No. (%) of isolates with discrepancies^d^** | | |
| --- | --- | --- | --- | --- | --- | --- | --- | --- | --- | --- | --- | --- | --- |
|  |  |  | **Range** | **MIC_50_** | **MIC_90_** |  | **S/WT** | **I/SDD** | **R/non-WT** |  | **VME** | **ME** | **MIE** |
| ***C. albicans* (n=35)** | | | | | | | | | | | | | |
| **5-flucytosine^e^** | **Vitek 2** | ND | ≤1 | ≤1 | ≤1 |  | NA | NA | NA |  |  |  |  |
|  | **ATB FUNGUS 3** |  | ≤4‒>16 | ≤4 | ≤4 | 34 (97.1) | 34 (97.1) | 1 (2.9) |  | NA | NA | NA | NA |
| **Amphotericin B** | **Vitek 2** | ECV: 2 | 0.5‒1 | 1 | 1 |  | 35 (100) |  |  |  |  |  |  |
|  | **ATB FUNGUS 3** |  | ≤0.5‒>16 | ≤0.5 | 4 | 32 (91.4) | 32 (91.4) |  | 3 (8.6) | 32 (91.4) |  | 3 (8.6) |  |
| **Fluconazole** | **Vitek 2** | S: ≤ 2, SDD: 4, R: ≥8 | ≤0.5‒16 | 1 | 8 |  | 19 (54.3) |  | 16 (45.7) |  |  |  |  |
|  | **ATB FUNGUS 3** |  | ≤1‒>128 | >128 | >128 | 7 (20) | 6 (17.1) | 2 (5.7) | 27 (77.2) | 20 (57.1) | 1 (2.9) | 12 (34.3) | 2 (5.7) |
| **Itraconazole** | **Vitek 2** | ND | NA | NA | NA |  | NA | NA | NA |  |  |  |  |
|  | **ATB FUNGUS 3** |  | ≤0.125‒>4 | >4 | >4 | NA | 5 (14.3) |  | 30 (85.7) | NA | NA | NA | NA |
| **Voriconazole** | **Vitek 2** | S: ≤ 0.12, I: 0.25-0.5, R: ≥ 1 | ≤0.12 | ≤0.12 | ≤0.12 |  | 35 (100) |  |  |  |  |  |  |
|  | **ATB FUNGUS 3** |  | ≤0.06‒>8 | >8 | >8 | 5 (14.3) | 5 (14.3) |  | 30 (85.7) | 5 (14.3) |  | 30 (85.7) |  |
| **Micafungin**  1 missed^h^ | **Vitek 2**  **(n = 34)** | S: ≤0.25, I: 0.5, R: ≥1 | ≤0.06 | ≤0.06 | ≤0.06 |  | 34 (97.1) |  |  |  |  |  |  |
|  | **ATB FUNGUS 3** |  | NA | NA | NA | NA | NA | NA | NA | NA | NA | NA | NA |
| **Caspofungin** | **Vitek 2** | S: ≤ 0.25, I: 0.5, R: ≥ 1 | ≤0.12‒0.25 | ≤0.12 | 0.25 | 34 (97.1) | 35 (100) |  |  | 34 (97.1) |  | NA |  |
|  | **E-test** |  | 0.125‒0.5 | 0.25 | 0.25 |  | 34 (97.1) | 1 (2.9) |  |  | NA | NA | NA |
| ***C. glabrata* (n=17)** | | | | | | | | | | | | | |
| **5-flucytosine^e^** | **Vitek 2** | ND | ≤1 | ≤1 | ≤1 |  | NA | NA | NA |  |  |  |  |
|  | **ATB FUNGUS 3** |  | ≤4‒>16 | ≤4 | ≤4 | 16 (94.1) | 16 (94.1) |  | 1 (5.9) | NA | NA | NA | NA |
| **Amphotericin B**  1 missed^h^ | **Vitek 2**  **(n = 16)** | ECV: 2 | ≤0.25‒4 | 0.5 | 0 |  | 14 (82.4) |  | 2 (11.8) |  |  |  |  |
|  | **ATB FUNGUS 3** |  | ≤0.5‒4 | 1 | 1 | 13 (76.5) | 15 (88.2) |  | 2 (11.8) | 12 (75) | 2 (12.5) | 2 (12.5) |  |
| **Fluconazole**  11 missed^h^ | **Vitek 2**  **(n = 6)** | SDD: ≤ 32, R: ≥ 64 | ≤0.5‒4 | ≤0.5 | 0 |  |  | 6 (35.3) |  |  |  |  |  |
|  | **ATB FUNGUS 3** |  | 16‒>128 | >128 | 0 | 0 (0) |  | 12 (70.6) | 5 (29.4) | 2 (33.3) |  |  | 4 (66.7) |
| **Itraconazole** | **Vitek 2** | ECV:4 | NA | NA | NA |  | NA | NA | NA |  |  |  |  |
|  | **ATB FUNGUS 3** |  | ≤0.125‒>4 | 2 | 0 | NA | 11 (64.7) |  | 6 (35.3) | NA | NA | NA | NA |
| **Voriconazole**  1 missed^h^ | **Vitek 2**  **(n = 16)** | ECV: 0.25 | ≤0.12‒0.25 | ≤0.12 | ≤0.12 |  | 16 (94.1) |  |  |  |  |  |  |
|  | **ATB FUNGUS 3** |  | 0.25‒>8 | 4 | >8 | 7 (43.8) | 8 (47.1) |  | 9 (52.9) | 7 (43.8) |  | 9 (56.2) |  |
| **Micafungin**  10 missed^h^ | **Vitek 2**  **(n = 7)** | S: ≤0.06, I: 0.12, R: ≥ 0.25 | ≤0.06 | ≤0.06 | ≤0.06 |  | 7 (41.2) |  |  |  |  |  |  |
|  | **ATB FUNGUS 3** |  | NA | NA | NA | NA | NA | NA | NA | NA | NA | NA | NA |
| **Caspofungin** | **Vitek 2** | S: ≤ 0.12, I: 0.25, R: ≥ 0.5 | 0.12‒8 | 0.5 | 4 | 14 (82.4) | 7 (41.2) | 1 (5.9) | 9 (52.9) | 15 (88.2) |  |  |  |
|  | **E-test** |  | 0.125‒>32 | 0.38 | 32 |  | 7 (41.2) | 1 (5.9) | 9 (52.9) |  | NA | NA | NA |
| ***C. tropicalis* (n=4)** | | | | | | | | | | | | | |
| **5-flucytosine^e^** | **Vitek 2** | ND | ≤1 | ≤1 | ≤1 |  | NA | NA | NA |  |  |  |  |
|  | **ATB FUNGUS 3** |  | ≤4 | ≤ 4 | ≤4 | 4 (100) | 4 (100) |  |  | NA | NA | NA | NA |
| **Amphotericin B** | **Vitek 2** | ECV: 2 | 0.5‒8 | 0.75 | 0 |  | 3 (75) |  | 1 (25) |  |  |  |  |
|  | **ATB FUNGUS 3** |  | ≤0.5‒2 | 1 | 2 | 4 (100) | 4 (100) |  |  | 3 (75) | 1 (25) |  |  |
| **Fluconazole** | **Vitek 2** | S: ≤ 2, SDD: 4, R: ≥8 | ≤0.5‒8 | 1 | 0 |  | 3 (75) |  | 1 (25) |  |  |  |  |
|  | **ATB FUNGUS 3** |  | ≤1‒64 | 16 | 32 | 2 (50) | 1 (25) | 1 (25) | 2 (50) | 1 (25) |  | 2 (50) | 1 (25) |
| **Itraconazole** | **Vitek 2** | ECV: 0.5 | NA | NA | NA |  | NA | NA | NA |  |  |  |  |
|  | **ATB FUNGUS 3** |  | 0.25‒>4 | 0.5 | 0 | NA | 2 (50) |  | 2 (50) | NA | NA | NA | NA |
| **Voriconazole** | **Vitek 2** | S: ≤ 0.12, I: 0.25-0.5, R: ≥ 1 | ≤0.12 | ≤0.12 | ≤0.12 |  | 4 (100) |  |  |  |  |  |  |
|  | **ATB FUNGUS 3** |  | ≤0.06‒>8 | 0.125 | 0 | 1 (25) | 1 (25) | 1 (25) | 2 (50) | 1 (25) |  | 2 (50) | 1 (25) |
| **Micafungin** | **Vitek 2** | S: ≤0.25, I: 0.5, R: ≥1 | ≤0.06‒0.25 | ≤0.06 | ≤0.06 |  | 4 (100) |  |  |  |  |  |  |
|  | **ATB FUNGUS 3** |  | NA | NA | NA | NA | NA | NA | NA | NA | NA | NA | NA |
| **Caspofungin** | **Vitek 2** | S: ≤ 0.25, I: 0.5, R: ≥ 1 | ≤0.12‒0.25 | ≤0.12 | 0 | 3 (75) | 4 (100) |  |  | 4 (100) |  |  |  |
|  | **E-test** |  | 0.125‒0.25 | 0.25 | 0 |  | 4 (100) |  |  |  | NA | NA | NA |
| ***C. guilliermondii* (n=2)** | | | | | | | | | | | | | |
| **5-flucytosine^e^** | **Vitek 2** | ND | ≤1 | ≤1 | ≤1 |  | NA | NA | NA |  |  |  |  |
|  | **ATB FUNGUS 3** |  | ≤4 | ≤4 | ≤4 | 2 (100) | 2 (100) |  |  | NA | NA | NA | NA |
| **Amphotericin B** | **Vitek 2** | ECV: 2 | 1‒8 | 4 | 0 |  | 1 (50) |  | 1 (50) |  |  |  |  |
|  | **ATB FUNGUS 3** |  | ≤0.5‒>16 | 8 | 0 | 0 (0) | 1 (50) |  | 1 (50) |  | 1 (50) | 1 (50) |  |
| **Fluconazole**  2 missed^h^ | **Vitek 2** | ECV: 1 | NA | NA | NA |  | NA | NA | NA |  |  |  |  |
|  | **ATB FUNGUS 3** |  | 16‒>128 | 32 | 0 | NA |  |  | 2 (100) | NA | NA | NA | NA |
| **Itraconazole** | **Vitek 2** | ECV: 2 | NA | NA | NA |  | NA | NA | NA |  |  |  |  |
|  | **ATB FUNGUS 3** |  | 1‒>4 | 2 | 0 | NA | 1 (50) |  | 1 (50) | NA | NA | NA | NA |
| **Voriconazole^e^** | **Vitek 2** | ND | ≤0.12 | ≤0.12 | ≤0.12 |  | NA | NA | NA |  |  |  |  |
|  | **ATB FUNGUS 3** |  | 0.125‒>8 | 4 | 0 | 1 (50) | 1 (50) |  | 1 (50) | NA | NA | NA | NA |
| **Micafungin** | **Vitek 2** | S: ≤ 2, I: 4, R: ≥ 8 | ≤0.06‒0.12 | ≤0.06 | ≤0.06 |  | 2 (100) |  |  |  |  |  |  |
|  | **ATB FUNGUS 3** |  | NA | NA | NA | NA | NA | NA | NA | NA | NA | NA | NA |
| **Caspofungin** | **Vitek 2** | S: ≤ 2, I: 4, R: ≥ 8 | ≤0.12‒0.25 | 0.18 | 0 | 0 (0) | 2 (100) |  |  | 0 (0) |  |  |  |
|  | **E-test** |  | 4 | 4 | 4 |  |  | 2 (100) |  |  | NA | NA | NA |
| ***C. kefyr* (n=1)** | | | | | | | | | | | | | |
| **5-flucytosinee** | **Vitek 2** | ND | ≤1 | ≤1 | ≤1 |  | NA | NA | NA |  |  |  |  |
|  | **ATB FUNGUS 3** |  | ≤4 | ≤4 | ≤4 | 1 (100) | 1 (100) |  |  | NA | NA | NA | NA |
| **Amphotericin B** | **Vitek 2** | ECV: 2 | 2 | 2 | 2 |  | 1 (100) |  |  |  |  |  |  |
|  | **ATB FUNGUS 3** |  | 1 | 1 | 1 | 1 (100) | 1 (100) |  |  | 1 (100) |  |  |  |
| **Fluconazole**  1 missed^h^ | **Vitek 2** | ECV: 8 | NA | NA | NA |  | NA | NA | NA |  |  |  |  |
|  | **ATB FUNGUS 3** |  | 16 | 16 | 16 | NA |  |  | 1 (100) | NA | NA | NA | NA |
| **Itraconazole** | **Vitek 2** | ECV: 0.5 | NA | NA | NA |  | NA | NA | NA |  |  |  |  |
|  | **ATB FUNGUS 3** |  | >4 | >4 | >4 | NA |  |  | 1 (100) | NA | NA | NA | NA |
| **Voriconazole^e^** | **Vitek 2** | ND | ≤0.12 | ≤0.12 | ≤0.12 |  | NA | NA | NA |  |  |  |  |
|  | **ATB FUNGUS 3** |  | >8 | >8 | >8 | 0 (0) |  |  | 1 (100) | NA | NA | NA | NA |
| **Micafungin**  1 missed^h^ | **Vitek 2** | ECV: 0.125 | NA | NA | NA |  | NA | NA | NA |  |  |  |  |
|  | **ATB FUNGUS 3** |  | NA | NA | NA | NA | NA | NA | NA | NA | NA | NA | NA |
| **Caspofungin^f^** | **Vitek 2** | ND | NA | NA | NA | NA |  |  |  | NA |  |  |  |
|  | **E-test** |  | 0.25 | 0.25 | 0.25 |  | NA | NA | NA |  | NA | NA | NA |
| ***C. parapsilosis* (n=1)** | | | | | | | | | | | | | |
| **5-flucytosine^e^** | **Vitek 2** | ND | ≤1 | ≤ 1 | ≤1 |  | NA | NA | NA |  |  |  |  |
|  | **ATB FUNGUS 3** |  | ≤4 | ≤4 | ≤4 | 1 (100) | 1 (100) |  |  | NA | NA | NA | NA |
| **Amphotericin B** | **Vitek 2** | ECV:1 | 0.5 | 0.5 | 0.5 |  | 1 (100) |  |  |  |  |  |  |
|  | **ATB FUNGUS 3** |  | ≤0.5 | ≤0.5 | ≤0.5 | 1 (100) | 1 (100) |  |  | 1 (100) |  |  |  |
| **Fluconazole** | **Vitek 2** | S: ≤ 2, SDD: 4, R: ≥8 | 8 | 8 | 8 |  |  |  | 1 (100) |  |  |  |  |
|  | **ATB FUNGUS 3** |  | >128 | >128 | >128 | 0 (0) |  |  | 1 (100) | 1 (100) |  |  |  |
| **Itraconazole** | **Vitek 2** | ECV: 0.5 | NA | NA | NA |  | NA | NA | NA |  |  |  |  |
|  | **ATB FUNGUS 3** |  | >4 | >4 | >4 | NA |  |  | 1 (100) | NA | NA | NA | NA |
| **Voriconazole** | **Vitek 2** | S: ≤ 0.12, I: 0.25-0.5, R: ≥ 1 | ≤0.12 | ≤0.12 | ≤0.12 |  | 1 (100) |  |  |  |  |  |  |
|  | **ATB FUNGUS 3** |  | >8 | >8 | >8 | 0 (0) |  |  | 1 (100) |  |  | 1 (100) |  |
| **Micafungin**  1 missed^h^ | **Vitek 2** | S: ≤ 2, I: 4, R: ≥ 8 | NA | NA | NA |  | NA | NA | NA |  |  |  |  |
|  | **ATB FUNGUS 3** |  | NA | NA | NA | NA | NA | NA | NA | NA | NA | NA | NA |
| **Caspofungin** | **Vitek 2** | S: ≤ 2, I: 4, R: ≥ 8 | 4 | 4 | 4 | 0 (0) |  | 1 (100) |  | 0 (0) |  |  |  |
|  | **E-test** |  | >32 | >32 | >32 |  |  |  | 1 (100) |  | NA | NA | NA |
| **Total isolates (n=60)** | | | | | | | | | | | | | |
| **5-flucytosine^e^** | **Vitek 2** |  | ≤1 | ≤1 | ≤1 |  | NA | NA | NA |  |  |  |  |
|  | **ATB FUNGUS 3** |  | ≤4‒>16 | ≤4 | ≤4 | 58 (96.7) | 58 (96.7) | 1 (1.7) | 1 (1.7) | NA | NA | NA | NA |
| **Amphotericin B**  1 missed^h^ | **Vitek 2**  **(n = 59)** |  | ≤0.25‒8 | 1 | 1 |  | 55 (91.7) |  | 4 (6.7) |  |  |  |  |
|  | **ATB FUNGUS 3** |  | ≤0.5‒>16 | ≤0.5 | 4 | 51 (86.4) | 54 (90) |  | 6 (10) | 49 (83.1) | 4 (6.7) | 6 (10.2) |  |
| **Fluconazole**  14 missed^h^ | **Vitek 2**  **(n = 46)** |  | ≤0.5‒16 | 1 | 8 |  | 22 (36.7) | 6 (10) | 18 (30) |  |  |  |  |
|  | **ATB FUNGUS 3** |  | ≤1‒>128 | 32 | >128 | 9 (19.6) | 7 (11.7) | 15 (25) | 38 (63.3) | 24 (52.2) | 1 (2.2) | 14 (30.4) | 7 (15.2) |
| **Itraconazole** | **Vitek 2** |  | NA | NA | NA |  | NA | NA | NA |  |  |  |  |
|  | **ATB FUNGUS 3** |  | ≤0.125‒>4 | >4 | >4 | NA | 19 (31.7) |  | 41 (68.3) | NA | NA | NA | NA |
| **Voriconazole**  1 missed^h^ | **Vitek 2^g^**  **(n = 56)** |  | ≤0.12‒0.25 | ≤0.12 | ≤0.12 |  | 56 (93.3) |  |  |  |  |  |  |
|  | **ATB FUNGUS 3** |  | ≤0.06‒>8 | 4 | >8 | 14 (23.7) | 15 (25) | 1 (1.7) | 44 (73.3) | 13 (23.2) |  | 42 (75) | 1 (1.8) |
| **Micafungin**  13 missed^h^ | **Vitek 2**  **(n = 47)** |  | ≤0.06‒0.12 | ≤0.06 | ≤0.06 |  | 47 (78.3) |  |  |  |  |  |  |
|  | **ATB FUNGUS 3** |  | NA | NA | NA | NA | NA | NA | NA | NA | NA | NA | NA |
| **Caspofungin**  1 missed^h^ | **Vitek 2**  **(n = 59)** |  | ≤0.12‒8 | ≤0.12 | 0.5 | 51 (86.4) | 48 (80) | 2 (3.3) | 9 (15) | 53 (89.8) |  |  |  |
|  | **E-test**  **(n = 59)** |  | 0.125‒>32 | 0.25 | 4 |  | 45 (75) | 4 (6.6) | 10 (16.7) |  | NA | NA | NA |

Abbreviations: CBPs, clinical breakpoints; ECVs, epidemiological cut-off values; MIC, minimum inhibitory concentration; EA, essential agreement; S, Susceptible; I, Intermediate; SDD, Susceptible dose-dependent; R, resistant; WT, wild type; non-WT, non-wild type; CA, categorical agreement; VME, very major error; ME, major error; MIE, minor error; ND, not defined; NA, not applicable.

^a^The species-drug combinations with no defined CBPs or ECVs were interpreted according to the manufacturer’s instructions of ATB FUNGUS 3.

^b^MIC_50_ and MIC_90_ were statistically expressed as the 50^th^ percentile (median) and the 90^th^ percentile, respectively.

^c^EA and CA were calculated based on the available results provided by the Vitek 2 method, and all missed data were excluded from the comparative analysis.

^d^The Vitek 2 method was not used as a gold standard for assessing caspofungin susceptibility, owing to the reported limitation with caspofungin.

^e^Since there are no CLSI CBPs or ECVs established for 5-flucytosine with all spp., voriconazole with *C. guilliermondii* and *C. kefyr*, CA was not calculated.

^f^MIC values obtained when testing caspofungin with *C. kefyr* were not reported, owing to the currently unavailable CBPs or ECVs.

^g^Three isolates (2 *C. guilliermondii* and 1 *C. kefyr*) were excluded, owing to the absence of CBPs or ECVs defined by the CLSI.

^h^Data was not given by the Vitek machine.
